# A Novel Role for Lh3 Dependent ECM Modifications during Neural Crest Cell Migration in Zebrafish

**DOI:** 10.1371/journal.pone.0054609

**Published:** 2013-01-18

**Authors:** Santanu Banerjee, Jesse Isaacman-Beck, Valerie A. Schneider, Michael Granato

**Affiliations:** Department of Cell and Developmental Biology, University of Pennsylvania School of Medicine, Philadelphia, Pennsylvania, United States of America; Instituto Gulbenkian de Ciência, Portugal

## Abstract

During vertebrate development, trunk neural crest cells delaminate along the entire length of the dorsal neural tube and initially migrate as a non-segmented sheet. As they enter the somites, neural crest cells rearrange into spatially restricted segmental streams. Extracellular matrix components are likely to play critical roles in this transition from a sheet-like to a stream-like mode of migration, yet the extracellular matrix components and their modifying enzymes critical for this transition are largely unknown. Here, we identified the glycosyltransferase Lh3, known to modify extracellular matrix components, and its presumptive substrate Collagen18A1, to provide extrinsic signals critical for neural crest cells to transition from a sheet-like migration behavior to migrating as a segmental stream. Using live cell imaging we show that in *lh3* null mutants, neural crest cells fail to transition from a sheet to a stream, and that they consequently enter the somites as multiple streams, or stall shortly after entering the somites. Moreover, we demonstrate that transgenic expression of *lh3* in a small subset of somitic cells adjacent to where neural crest cells switch from sheet to stream migration restores segmental neural crest cell migration. Finally, we show that knockdown of the presumptive Lh3 substrate Collagen18A1 recapitulates the neural crest cell migration defects observed in *lh3* mutants, consistent with the notion that Lh3 exerts its effect on neural crest cell migration by regulating post-translational modifications of Collagen18A1. Together these data suggest that Lh3–Collagen18A1 dependent ECM modifications regulate the transition of trunk neural crest cells from a non-segmental sheet like migration mode to a segmental stream migration mode.

## Introduction

During vertebrate development, trunk neural crest cells delaminate along the entire rostro-caudal axis from the neural tube, and then continue along specific migratory routes [1]. Neural crest cells that delaminate later during development enter a ventro-lateral pathway between the dermomyotome and the epidermis [2], [3]. Early delaminating neural crest cells choose a ventral route through the intersomitic space along intersegmental blood vessels, and between the somites and the neural tube along a pathway called the ventro-medial pathway [4]. Importantly, neural crest cells entering the ventromedial pathway converge from a broader region along the neural tube into distinct segmentally organized streams. In amniotes and rodents, these streams extend only in the rostral portion of the somite where they subsequently travel alongside with ventrally projecting spinal nerves [5], [6], [7]. Ablation experiments in zebrafish have shown that segmental neural crest cell migration can occur independent of spinal motor axons, suggesting that other cues direct this migration [8].

It is well established that somite derived signals direct segmental neural crest cell migration [9], [10]. These include Ephrin/Eph receptor dependent signals [11], [12], [13], Semaphorin3-Neuropilin dependent signals [14], [15], [16] and Wnt-MuSK dependent signals [8], all thought to provide inhibitory and/or repulsive forces to restrict neural crest cells migration to a defined region of the somite. Although several extracelluar matrix (ECM) components have been shown to localize along the segmental path where they might exert permissive, pro migratory roles [17], ECM components required for specific aspects of segmental neural cell migration *in vivo* have not been identified. Similarly, the enzymes that modify ECM components post-translationally and thereby provide them with unique properties to regulate neural crest cell behaviors are largely unknown. Zebrafish provide an attractive system to identify the role of ECM components and their modifications for neural crest cell migration [18].

In zebrafish, neural crest cells migrate through a restricted region of the somite located mid-segmentally between two adjacent somite/segment boundaries [19], [20]. We have recently shown that the secreted glycoprotein Wnt11r binds the Muscle specific kinase (MuSK) to induce a Dishevelled dependent signaling cascade in adaxial muscle cells. In embryos compromised for Wnt11r-MuSK-Dishevelled signaling, neural crest cells stray away from the mid-segmental region [8]. However, in these embryos, the segregation of neural crest cells into mid-segmental streams remains unaffected. This observation raised the possibility that additional, somite derived signaling pathways regulate the transition of neural crest cells migrating as a sheet into segmentally repeated streams.

Lh3 (Lysyl hydroxylase 3, or 2-oxoglutarate 5-dioxygenase 3 PLOD3), is a multifunctional enzyme that catalyzes the post-translational addition of galactosyl and glucosyl moieties onto collagens and other proteins with collagen-like domains [21]. Collagens, depending on their particular subtypes, can function as either permissive or non permissive substrates for neural crest cells, however the full complement of their *in vivo* roles for neural crest cell migration are not well defined [17]. Here, we present genetic evidence for a Lh3 dependent signaling pathway that acts cell-non autonomously for neural crest cells to transition from a sheet-like to a stream-like mode of migration. In *lh3* mutant embryos neural crest cells fail to transition, leading to ectopic or stalled migration. We demonstrate that these neural crest cell migration defects occur independently of the motor axon guidance defects observed in *lh3* mutants, and show that *lh3* activity in a subset of muscles cells is sufficient for proper neural crest cell migration. Finally, we show that morpholino mediated knockdown of *collagen18a1* leads to neural crest cell migration defects similar to those observe in *lh3* mutants. We propose that Lh3 dependent ECM modifications, including those via Collagen18A1, regulate the behavioral transition of trunk neural crest cells from a non-segmental, sheet like mode to a segmental, stream like mode.

## Results

### Lh3 is Required for Segmental Neural Crest Cell Migration

**Figure 1:**
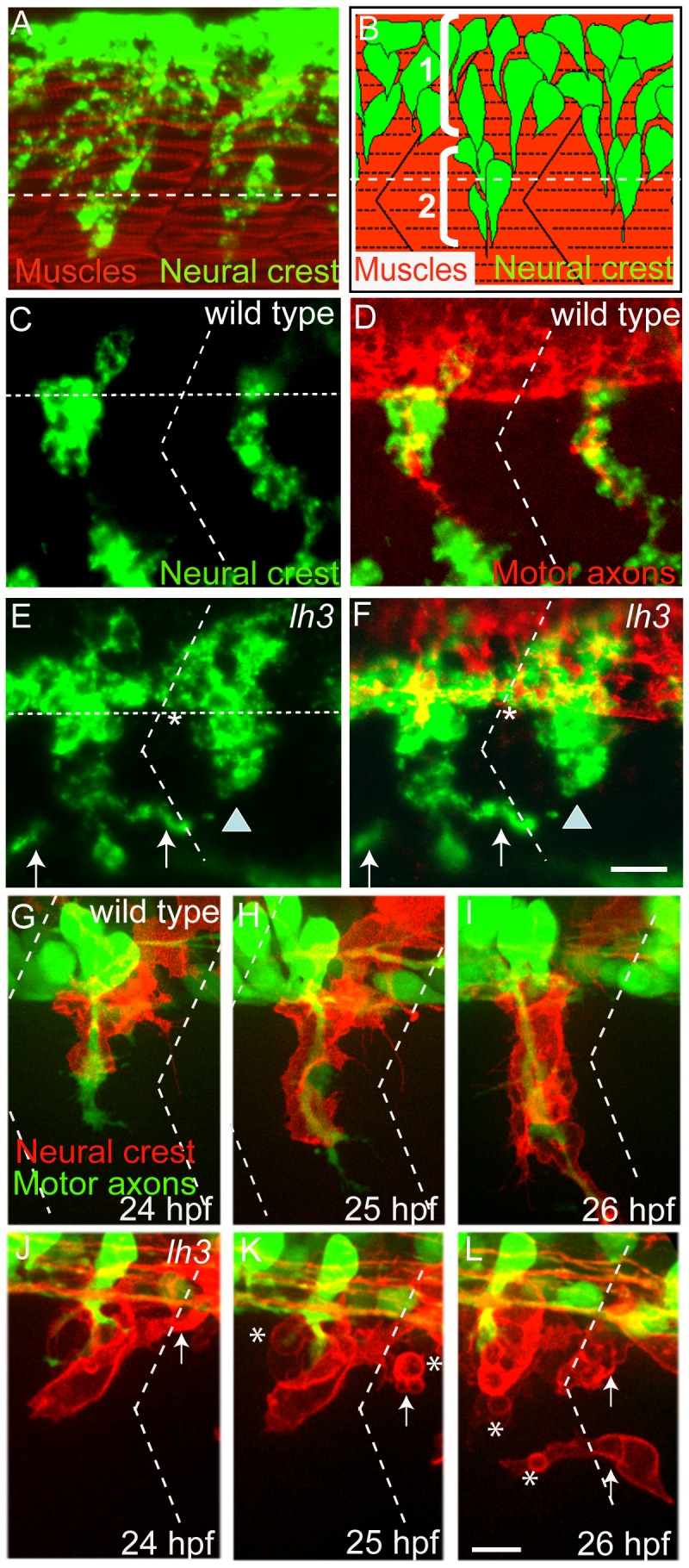
The role of *lh3* signaling in neural crest cell migration. (A) Lateral view of a 22 hour old embryo stained with the pan-neural crest cell marker *crestin* (riboprobe, in green) marking neural crest cells, and with F59 antibody marking adaxial muscle cells. (B) Schematic diagram showing two modes of zebrafish neural crest cell migration: 1) sheet-like, non segmental and 2) stream-like, segmental. Neural crest cells (green) migrate through a central region of each somite after forming segmental streams (red). White horizontal dashed lines in A, B, C and E indicate approximate position of the ventral boundary of the neural tube. (C and D) Lateral view of a 28 hpf embryo stained with *crestin* (green) and the motor neuron maker antibodies (Znp1 and SV2; red). Horizontal dotted lines in A indicate the ventral boundary of the neural tube. E and F show lateral views of *lh3* mutant embryos stained with *crestin* and motor neuron markers. Arrows in E and F point to neural crest cells near the somite boundaries. Asterisks marks neural crest cells forming sheet between two adjacent hemisegments. G–L shows still images from time lapse movies recorded from embryos expressing membrane bound RFP (Tg[*sox10*:*mrfp*]) in neural crest cells and GFP in motor neurons (Tg[*mnx1:gfp*]). In wildtype embryos (G–I), neural crest cells always migrate mid-segmentally along with motor axons. In *lh3* mutant embryos (J–L), neural crest cells migrate along with motor axons mid-segmentally and in the region near segment/somite boundary (arrows). Neural crest cells in *lh3* mutants also acquire a rounded morphology (asterisks). Oblique dotted lines in C to L indicate position of the somite/segment boundary. Scale bars:10 micron.

In zebrafish neural crest cells delaminate from the dorsal neural tube and enter the ventro-medial pathway as an uninterrupted, non-segmented group of cells (Fig. 1 A and B) [20]. Given its sheet like appearance previously described in birds and mammals, we will refer to this mode of non-segmented migration as sheet like migration [22]. At the interface between neural tube and notochord, neural crest cells converge into discreet segmental streams, each stream migrating ventrally along a narrow path in the center of each somite (Fig. 1 A, C, D). We will refer to these individual streams as ‘mid-segmental’ streams. As they continue their migration, neural crest cell streams share the path with spinal motor axons (Fig. 1 C and D).

In *lh3^tv205/tv205^* mutants (referred as *lh3* hereafter), neural crest cells delaminated properly from the dorsal neural tube and migrated ventrally. However, at the interface between neural tube and notochord, where wildtype neural crest cells converge into discreet mid-segmental stream, a significant fraction of *lh3* neural crest cells failed to coalesce into segmental streams and instead remained organized in a continuous sheet along the anterior posterior axis (asterisks in Fig. 1E, F asterisks; 25%, n = 80 hemisegments). Although many *lh3* mutant neural crest cells entered the mid-segmental path, they either stalled shortly after entering the path (arrowheads in Figure 1E, F 50%, n = 80 hemisegments), or they migrated through lateral somite territories typically devoid of neural crest cells (arrows in Fig. 1E, F; 25%, n = 80 hemisegments).

Based on the static images we were unable to determine whether these mutant neural crest cells in the lateral somite entered the midsegmental path but then strayed away or whether they never entered the mid-segmental stream and instead entered the somites following an ectopic route. To distinguish between these two possibilities, we performed live imaging of wildtype (*lh3* siblings) and *lh3* mutant embryos expressing membrane bound RFP in neural crest cells (Tg[*sox10*:*mRFP*]*^vu234^*) and GFP in motor neurons (Tg[*mnx1*:*gfp*]*^ml2^*). In wildtype embryos neural crest cells migrate as a single stream in the mid-segmental region, remaining in close association with motor axons (Fig. 1 G–I). In contrast, analysis of *lh3* mutant embryos revealed one or both of the following migration defects (10/11 somitic segments from 4 embryos). In 6/10 affected somite segments, *lh3* mutant neural crest cells correctly coalesced into a mid-segmental stream, but eventually stalled (data not show), consistent with our end-point analyses (Fig. 1E). In 4 out of 11 somite segments analyzed, *lh3* mutant neural crest cells also entered the somite along an ectopic route, lateral to the mid-segmental path (Fig. 1J, arrow). Over the next few hours these misguided neural crest cells continued on their ectopic path in close proximity to the somite boundary (Fig. 1K, L, arrows), a path never observed in wildtype embryos. Finally, we noticed that in *lh3* mutants individual neural crest cells frequently lacked filopodial protrusions, characteristic for wildtype neural crest cells within the mid-segmental stream. Instead, *lh3* mutant neural crest cells exhibited a more round morphology (Fig. 1K, L, asterisks). Thus, in *lh3* mutants neural crest cells either enter the mid-segmental path but then stall, or fail to organize into mid-segmental streams. Instead, mutant cells initially accumulate where wildtype cells transition into segmental streams, and then frequently enter into the somites along the entire length of the somite, forming ectopic, non-segmental streams.

### Neural Crest Cell Defects in *lh3* Mutants Occur Independent of Motor Axons

Along their ventro-medial route through the somites, zebrafish neural crest cells share a migration path with spinal motor axons [8], [23]. We have previously shown that while misguided motor axons can redirect neural crest cells, motor axons are dispensable for neural crest cells to enter and migrate into the mid-segmental path [8]. In *lh3* mutants, many motor axons fail to exit the spinal cord, and those that exit stall soon thereafter [24]. To determine if neural crest cell migration defects observed in *lh3* mutants are caused by motor axons failing to migrate properly, we examined *lh3* mutant neural crest cell migration following motor neuron ablation.

**Figure 2:**
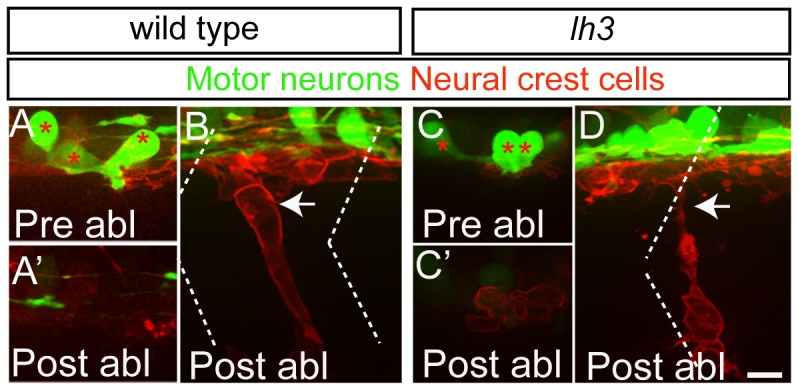
Motor axon independent role of *lh3* signaling in neural crest cell migration. (A and D) Lateral view of double transgenic embryos (Tg[*sox10:mrfp*];Tg[*mnx1;gfp*] expressing mRFP in neural crest cells and GFP in neurons. Pre ablation (A) and post ablation (A’) view of motor neurons and neural crest cells in wildtype embryos. Neural crest cells were able to migrate through the mid-segmental region 4 hours following motor neuron ablation (B). In *lh3* mutants (C- pre ablation, C’−30 min post ablation, D- 4 hours post ablation), neural crest cells migrated in the region overlying somite/segment boundary in the absence of motor neurons. Scale bar- 10 micron.

As previously reported, in the absence of motor neurons wildtype neural crest cells enter and migrate along the mid-segmental path (Fig. 2 A–A’’–B; n = 4/7). Ablation of motor neurons in *lh3* mutants did neither ameliorate nor enhance the neural crest cell migration defects observed in *lh3* mutants. In motor neuron ablated *lh3* mutants, neural crest cells frequently entered through the mid-segmental path (n = 4/10; Fig. 2 C–C’’–D), and stalled, or entered and extended through the somites along ectopic, non-segmental routes (n = 3/10). Thus, failure of *lh3* mutant neural crest cells to coalesce into segmental streams is independent of motor axons. This suggests that the ability of neural crest cells to converge into a single mid-segmental stream is directly dependent on *lh3* activity, either intrinsically within migratory neural crest cell, or provided extrinsically by the embryonic environment.

### Lh3 Function in Adaxial Muscle for Neural Crest Cell Convergence

**Figure 3:**
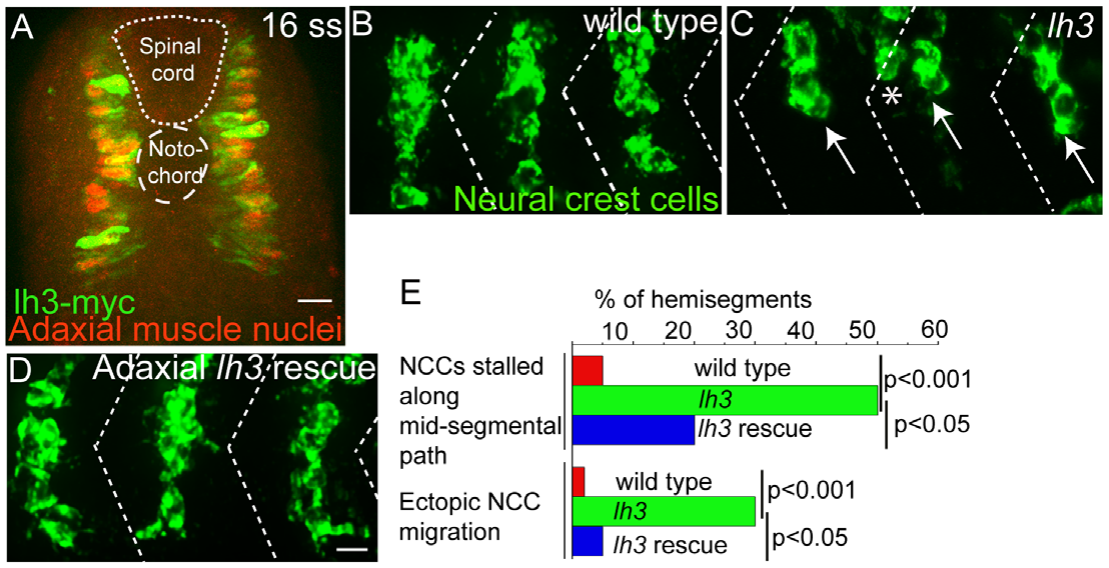
Lh3 activity in adaxial cells can rescue neural crest cell migration defects in *lh3* mutants . Cross sectional view of 16 somite stage [Tg(*smhc1*:*lh3-myc*)] embryos showing expression of lh3-myc transgene (green) in prox-1 (red) positive adaxial cells. Lateral view of an 28 hour old *lh3* sibling embryo (B), *lh3* mutant (C) and an *lh3* mutant embryo expressing *lh3-myc* in adaxial muscle cells (D), all stained with *crestin* (green). Note the stalled neural crest cells in the mid-segmental and neural crest cells in the hemisegment region in *lh3* mutants (arrows and asterisks in C). These defects were rescued in by expressing *lh3-myc* in adaxial cells of *lh3* mutants (C). (E) Quantification of neural crest cell migration defects. P values (** p<0.001; * p<0.05) were obtained using Chi- Square test. Scale bar-10 micron.

During the time period of neural crest cell migration, *lh3* mRNA is detectable in the notochord and in a small subpopulation of somitic muscle cells known as adaxial cells [25]. These adaxial cells decorate the future mid-segmental path, and genetic studies have shown that in the absence of adaxial cells, neural crest cells fail to form segmental streams [26]. Therefore, we asked if *lh3* activity in adaxial cells is functionally important for neural crest cells to organize into segmental streams. To test this, we generated transgenic zebrafish lines expressing Myc epitope tagged full length wildtype *lh3* cDNA under the control of an adaxial muscle specific promoter [27] (Tg[*smhc1*: *lh3-myc*]*^p161^*). In transgenic Tg[*smhc1*: *lh3-myc*]*^p161^* embryos *lh3-myc* expression is detectable in adaxial cells during the time period when neural crest cells begin to migrate along the ventro-medial pathway (Fig. 3A). Although levels of *lh3-myc* expression vary between individual adaxial muscle cells, we confirmed that over 90% of adaxial muscle cells, identified by the expression of Prox-1 [28], [29], also express *lh3-myc* (Fig. 3A). This transgenic line was crossed into *lh3* mutants, and neural crest cell migration was analyzed in the offspring from intercrosses between (*lh3*/+; Tg[*smhc1*:*lh3-myc*]) animals. Unlike wildtype embryos in which neural crest cells always entered and migrated through the somites along the mid-segmental path, in *lh3* mutant embryos neural crest cells either entered the mid-segmental path and then stalled (∼50% of hemisegments, n = 60; Fig. 3C, arrow), or they entered into and migrated through the somites along an ectopic, non-segmental path (∼30% of hemisegments, n = 60; Fig. 3C, asterisk). In contrast, transgenic expression of *lh3-myc* in adaxial muscle restored neural crest cell migration significantly in otherwise *lh3* mutant embryos (Fig. 3D, quantified in E). In these embryos, ectopic, non-segmental neural crest cell migration was restored to almost wildtype levels, suggesting that adaxial *lh3* expression regulates primarily the transition of trunk neural crest cells from a non-segmental sheet-like migration mode to a mid-segmental stream migration mode. Thus, *lh3* regulates cell non-autonomously the conversion form a sheet-like, non-segmental to segmental neural crest cell migration.

### Collagen18A1 Plays a Role in Neural Crest Cell Migration

**Figure 4:**
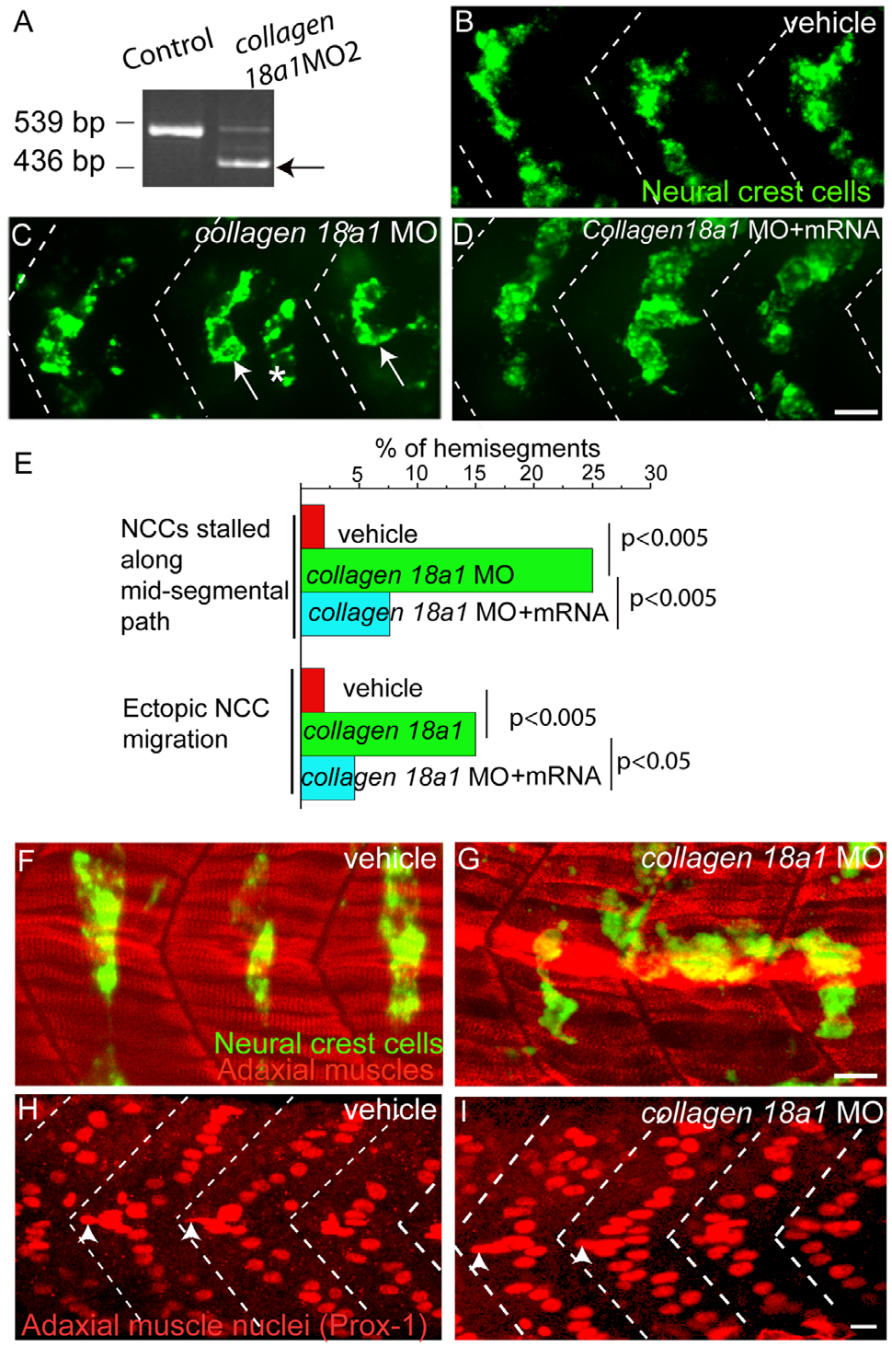
Knockdown of *collagen18a1* results in neural crest cell migration defects. (A) RT PCR analysis showing efficiency of *collagen18a1* knockdown following morpholino treatment. Arrow marks the expected band following morpholino treatment sized at 439 base pairs. Lateral views of 28 hpf vehicle (B) *collagen18a1* MO (C) and *collagen18a1* MO plus *collagen18a1* mRNA injected embryo (D), stained with *crestin* to visualize neural crest cells. Arrows indicate neural crest cells stalled along the mid-segmental path (C), and asterisks indicate neural crest cells along the ectopic path. (C, E) (E) Quantification of neural crest cell migration defects in *collagen18a1* MO injected embryos. p values were calculated using one tailed Fisher Exact Probability test. Vehicle injected and *collagen18a1*(G) MO injected embryos, stained with F59 to visualize adaxial cells (red), and *crestin* to visualize neural crest cells (green) (F–G) and with prox-1 antibody (H–I). Arrowheads mark adaxial cell nuclei located anteriorly near horizontal myoseptum region (H–I). Scale bar-10 micron.

We next examined how *lh3* influences neural crest cell migration. Lh3 is a multifunctional enzyme that catalyzes the post-translational addition of glycosyl moieties primarily onto collagens [21], thereby modifying the extracellular matrix. We had previously shown that early during neural crest cell migration, *lh3* and *collagen18a1* are co-expressed in adaxial muscle cells [25], suggesting a model by which Lh3 might influence neural crest cell migration via post-translational modifications of Collagen18A1. Importantly, Collagen18A1 belongs to the multiplexin group of non-fibrillar collagens and contains a cysteine rich domain (CRD) homologous to that of WNT binding *frizzled* receptors, and a unique thrombospondin (TSPN) domain, commonly found in the ectodomain of semaphorins and other guidance receptors [30]. Moreover, *collagen18a1* has previously shown to be critical for axon guidance in *C.elegnas*, *Drosophila* and zebrafish [25], [31], [32]. Therefore, we asked whether *collagen 18a1* function is important for neural crest cell migration. For this we used a combination of two splice blocking morpholinos (MOs) to knock down *collagen18a1* function. We had previously shown that MO1 blocks splicing of *collagen18a1* mRNA [25]. We also designed a second spice blocking MO, MO2, which also was effective in reducing *collegen18a1* transcript level in injected embryos (Fig. 4A). In embryos injected with vehicle (Danio buffer) solution, migration pattern of neural crest cells were indistinguishable from those observed in wild type embryos (Fig. 4 B). In embryos injected with a combination of *collagen18a1* MO1 and MO2 at a dose causing minimal lethality (<10%) and no obvious morphological defects, neural crest cell migration defects were almost identical to those observed in *lh3* mutants. Specifically, in *collagen18a1* MO1 and MO2 injected embryos neural crest cells either entered the mid-segmental path but then stalled (∼25%, n = 80 hemisegments; arrows in Fig. 4C, quantified in Fig. 4E), or they entered into and migrated through the somites along an ectopic, non-segmental path (∼15%, n = 80 hemisegments; asterisks in Fig. 4C, quantified in Fig. 4E).

Importantly, *collagen18a1* MO injection did not significantly affect adaxial cell morphology or differentiation (Fig. 4F, G). Further analyses using the adaxial cell specific Prox-1 marker [28], [29] revealed that adaxial cell number was unchanged in *collagen18a1* MO injected embryos (Fig. 4 H and I). Moreover, in wildtype embryos a subset of adaxial cell nuclei at the level of the horizontal myoseptum are located close to the anterior somite boundary, and their stereotyped position was unaffected in *collagen18a1* morpholino injected embryos (arrowheads, Fig. 4 H and I). Finally, we co-injected MO1 and MO2 and then synthetic mRNA encoding full-length *collagen18a1*. In these double injected embryos, neural crest migration was restored to almost wildtype levels, confirming that the neural crest cell phenotypes observed in *collagen18a1* MO injected embryos are caused by knockdown of *collagen18a1* function (Fig 4 D, quantified in E). Thus, knockdown of *collagen18a1* recapitulates the neural crest migration defects observed in *lh3* mutants, albeit with lower penetrance, suggesting that Lh3 acts in part through Collagen18A1 to guide neural crest cell migration.

## Discussion

Segmental migration of neural crest cells is dependent on a number of well studied guidance cues which act via their cognate receptors present on neural crest cells, and on less well characterized ECM components deposited along the migration path [17]. For example, in mammals, Nrp2-Sema3F signaling has been shown to organize neural crest cells into segmental streams [14], [15]. Although the distribution of ECM components along the migratory path of neural crest cells has been suggestive of specific functions for their migration, which and how ECM components and their modifications alter migratory patterns of neural crest cells has been unclear. Here we use genetic mutants, live cell imaging and transgenic rescue to examine the function of defined ECM components and ECM modifying enzymes for the migration of zebrafish trunk neural crest cells. Specifically, we show that in *lh3* mutants and *collagen18a* morphants neural crest cells frequently enter but fail to migrate along the mid-segmental path into the somites, and instead enter into and continue to migrate through somite territories typically devoid of neural crest cells. Moreover, we show that Lh3 activity is provided by a small group of somitic cells, the adaxial muscle cells, positioned where neural crest cells organize into segmental streams. Combined, our data identify an Lh3-Collagen18A1 dependent pathway that provides extrinsic signals required to alter the migratory behavior of neural crest cells as they organize into segmental streams.

### Lh3 Plays a Novel, Motor Axon Independent Function for Neural Crest Cell Migration

Neural crest cells, following delamination from the neural tube enter one of two migratory pathways, known as the dorso-lateral and the ventro-medial pathway, respectively. Along the ventromedial pathway neural crest cells undergo an intriguing re-organization as they transform from a non-segmented sheet of cells into distinct and segmented streams, which then continue their migration through the center of each somite. In *lh3* mutants, neural crest cells either enter through the mid-segmental path but then stall, or ignore the mid-segmental path and enter the somites at ectopic positions, demonstrating that *lh3* is critical for segmental neural crest cell migration.

In zebrafish, neural crest cells undergo the transition from a non-segmented sheet of cells into distinct and segmented streams at the ventral extend of spinal cord, adjacent to adaxial cells (Fig. 1A and B). Transgenic expression of *lh3* in adaxial cells rescued both migratory defects, stalling along the segmental path and migration along ectopic trajectories, albeit to a different extent. Although we can not exclude the possibility that the adaxial cell specific transgene produces Lh3 protein at levels below those produced endogenously by adaxial cells, the difference in the rescue levels is consistent with the idea that Lh3 exhibits two distinct functions for neural crest cell migration. First, an adaxial cell dependent function that provides extrinsic signals to re-organize migration of neural crest cells from a non-segmented sheet of cells into distinct and segmented streams. Secondly, Lh3 functions to support or promote neural crest cell migration along the segmental path, although this function appears to be provided only partially by adaxial cells, as transgenic expression failed to fully restore migration along the segmental path. Importantly, both functions appear independent of the presence of motor axons (Fig. 2) and both functions require, at least in part, Collagen18A1 function.

### Collagen18A1, a Multifunctional ECM Component and Presumptive Lh3 Substrate is Important for Segmental Neural Crest Cell Migration

One possible scenario by which Lh3 regulates neural crest cell migration is through the modification of ECM components. Lh3 is an ER resident glycosylating enzyme that adds O-linked glycosyl residues to collagens and collagen domain containing proteins [21]. Although *Lh3* as been detected in the extracellular space [33], we have previously shown that removing the ER retention signal from the *lh3* coding sequence abolishes *lh3*’s ability to restore motor axon guidance. We did not examined if a secreted form of Lh3 rescues neural crest cell migration, but favor the idea that Lh3 acts through several substrates, including Collagen18A1.

The best characterized targets of Lh3 are collagens [34], and we have previously shown that zebrafish Collagen18A1, a member of the multiplexin family of collagens, is transiently expressed in adaxial cells during the time of neural crest cell migration [25]. Knockdown of *collagen18a1* results in a neural crest cell migration defects similar to those observed in *lh3* mutant embryos, suggesting that Lh3 might indeed exert its function for neural crest cell migration by post-translational modifications of Collagen18A1. Importantly, the severity of neural crest cell migration defects observed in *collagen18a1* morphants is lower compared to those observed in *lh3* mutants. This may be simply due to residual *collagen18a1* function in morphant embryos. Alternatively, it is possible that Collagen18A1 is only one of several substrates through which Lh3 mediates neural crest cell migration.

### How does Lh3 and Collagen18A1 Influence Neural Crest Cell Migration?

There are several potential mechanisms through which Collagen18A1 might influence segmental neural crest cell migration. First, at the C terminus Collagen18A1 contains an endostatin domain previously shown to inhibit epithelial cell migration [35]. It is therefore conceivable that Lh3 modification of Collagen18A1 generates non-permissive territories to either sides of the mid-segmental path, thereby inhibiting neural crest cells to stray from the mid-segmental path. Loss of *lh3* or *collagen18a1* would make these territories permissive to neural crest cells, thereby resulting in ectopic migratory streams. However, such inhibitory migratory function of Collagen18A1 is less compatible with the frequent stalling of neural crest cells along the mid-segmental path observed in *collagen18a1* morphants and *lh3* mutants. Thus, Collagen18A1 might simultaneously promote neural crest cell migration, similar to that documented for neuronal migration [31].

Second, Collagen18A1 might influence neural crest cell migration through interactions with known positive or negative migratory cues. For example, Collagen18A1 might influence neural crest cell migration through its WNT binding cysteine rich domain (CRD) and/or its thrombospondin domain, commonly found in the ectodomains of semaphorin guidance cues [30]. Alternatively, Collagen18A1 might bind through the thrombospondin domain to Integrin receptors present on neural crest cells, thereby providing a permissive substrate for their migration [36]. Future structure-function studies to identify which Collagen18A1 domain(s) are required for neural crest cell migration will greatly aid to reveal the molecular mechanisms by which Collagen18A1 exerts its function on migrating neural crest cells.

Independent of the precise mechanism by which *Collagen18A1* influences neural crest cell migration, our data are consistent with a model by which Lh3 activity in adaxial muscle cells modifies ECM components which in turn influence cytoskeletal remodeling of neural crest cells during their migration. Recently, Lh3 has been shown to regulate the organization of extracellular matrix components such as Fibronectin and Collagens [37]. Consequently, such ECM modifications can induce changes in the cytoskeletal dynamics and in the appearance of motile cells. Consistent with this, fibroblasts derived from *lh3* deficient mice exhibit a more round morphology [37], similar to the morphology we observe in neural crest cells in *lh3* mutants and *collagen18a1* morphants. Thus, it is conceivable that *lh3* expression in adaxial cells regulates the modification and distribution of ECM components, including Collagen18A1, thereby providing extrinsic signals required to alter the migratory behavior of neural crest cells as they organize into segmental streams.

## Materials and Methods

### Ethics Statement

All experiments were conducted according to an Animal Protocol fully approved by the University of Pennsylvania Institutional Animal Care and Use Committee (IACUC) on September 7, 2010, protocol number 803258.Veterinary care is under the supervision of the University Laboratory Animal Resources (ULAR) of the University of Pennsylvania.

### Zebrafish Genetics

Zebrafish embryos used in this study were raised at 28°C for the required amount of time (see [38]). Wildtype fish used for experiments were TLF, and mutants used are *diwanka/lh3^tv205^*, [39], Tg[*mnx1:gfp*]*^ml2^* [40], Tg[*Sox10:mrfp*]*^vu234^* [41],Tg[*smhc1:lh3-myc*]*^p161^.*Transgenic fish were either used alone, in combination with each other or in combination with *lh3^tv205^* mutant background.

### Molecular Biology

*lh3*-*5x myc (*referred as *lh3-myc)* was cloned previously in pCS2+ vector [25]. *lh3*-*5x myc* was cloned in ISce-I vector downstream of smhc1 promoter using Xba/Spe1 sites.

### Anti Sense Morpholino Analysis

*collagen18a1* Morpholino 1∶5′ - TAACAACCTACCTGGATAGAGCCTT - 3′ [25]. *collagen18a1* Morpholino 2∶5′ - CAGTGCTCAACACACCTTGTCTCC - 3′ (This is a spice blocking morpholino that interferes with transcription between Exon 8 and Intron 8 of the CRD containing variant of *collagen18a1*). A total of 7 ng (MO1-5 ng; MO2- 2 ng) of *collagen18a1* MO cocktail was injected in every embryo. For RT PCR analysis, 5 uninjected and 5 morpholino-2 treated embryos were used to make cDNA. PCR were performed using a forward primer in Exon 5 (5′-GTGTTATTGGTGATCTGAGGGTGT-3′) and a reverse primer in Exon 10 (5′-TCCTCTTGGTCCTGTGACTTTCTG-3′) of the *collagen18a1* gene.

### Cloning of *collagen18a1* cDNA

A 1.5 kb fragment of *collagen18a1* cDNA containing sequence from Exon 4 to the stop codon of *collagen 18a1* were cloned into TOPO blunt vector named Topo-C183’-end using following primers (forward 5′CACTCAAGAATGAGATGAAGGGTG3′ and reverse TTCTAGATTGGC GTTACGGCGAATAGA). A 5′ 3 kb fragment of *collagen18a1* encompassing UTR and Exon 1-2 were cloned in pcrBluntII-TOPO-C18Ex1-2-5′ using the following primers (forward 5′ AATCGATACCATGG CAAGAAGGTGTCTCG and revere 5′ GGACCTGGAGGTCCAGGAATACTGAG). pcrBluntII-TOPO-C18 Exon1-2-5′ fragments were then digested with Sal1-Xho1 sites to release a 3 kb fragment which was subsequently moved into Sal1-Xho1 digested TopoC183’end vector to generate TOPOC18Exon1-2. A 4.5 kb fragments were then generated using Cla1 and Xb1 sites to clone into pCS2 vector generating pCS2C18Ex1-2 lacking exon 3 of *collagen18a1*. The Exon 3 of *collagen18a1* containing CRD domain (1.5 Kb) were subcloned into TOPOC18Ex3CRD5′ using primers (forward 5′ AATCGATA CCATGATGGCTGAACTCAGGT and reverse 5′TGCTCTCCATCT CCACTTGCTCC GTAAT). This fragment was then subcloned into pCS2C18Ex1-2 using Cla1- Xba1 site generating full length *Collagen18a1* cDNA (pCS2C18Ex3CRD). Finally, a 5X myc tag was cloned beyond the signal peptide via a newly created unique NheI site to track transgenic expression. Briefly, a unique Nhe-1 site was created by mutating C696T using the QuikChange II XL Site-Directed Mutagenesis Kit and the following primers: 5′TTGAAAGCCAATTTTTAATAGCTAGCATGCCAGCATCC TTC3′ (forward) and 5′GAAGGATGCTGGCATGCTAGCTATTAAAAATTGGCTTT CAA3′ (reverse). A 5X myc tag lacking a stop codon was then amplified from ISceI-smyhc1:SV1-myc [42] with primers flanked by NheI sites: (forward 5′AAAAGCTAGCCGTAA GGTAAATCGATCGAAA3′ and reverse 5′AAAAGCT AGCGGTGAGGTCGCCCTTGCTCTC3′). The amplified product was digested with NheI and subcloned into pCS2C18Ex3CRD to generate full length myc tagged *collagen18a1* cDNA into pCS2C18Ex3CRD-myc.

### mRNA Preparation and Injection

Full length myc tagged *collagen18a1* mRNA was made from pCS2C18Ex3CRD-myc vector through *in vitro* transcription using the Ambion mMESSAGE mMachine SP6 kit (Product # AM1340). A 500 ng/uL working stock was diluted in 0.1M KCl in DEPC water and a final concentration of 20 pg were injected per embryo.

### Immunohistochemistry

Antibody staining was performed as described previously [43]. The following primary antibodies were used: znp-1 (1∶200) [44]; Antibody Facility, University of Oregon; SV2 (1∶50, Developmental Studies Hybridoma Bank, University of Iowa), myc (9E10, 1∶1000,Covance), GFP (JL-8, 1-100,Clonetech), prox-1 (1∶2000). Antibodies were visualized with Alexa-Fluor-594 conjugated secondary antibodies (1∶500; Molecular Probes, Eugene,OR). In situ hybridization with *crestin* probe [45] was performed as described in [25].

### Live Cell Imaging

Sixteen to twenty somite stages embryos were briefly anesthetized using tricane and then mounted laterally in 1% low melting agarose prepared in Ringer’s solution containing tricane. Images were captured in 1–10 min interval using 63x water immersion lens in a spinning disc confocal microscope (Olympus) equipped with a 28°C temperature controlled chamber. Appropriate numbers of z sections were used to create maximum intensity projection image using Slidebook (3i) or NIH ImageJ. Images were further processed using ImageJ and/or Photoshop.

### Motor Neuron Ablation

Embryos were mounted as described for live imaging. Motor neuron cell bodies expressing GFP were ablated using a MicroPoint nitrogen pulsed laser (Photonic Instruments) mounted on a spinning disc microscope with a 63x water immersion objective lens. Ablations were carried out in up to four hemisegments per embryo. Ablations were verified after 30 minutes, and after 3 hours. Segments with incomplete/partial ablation of motor neurons were not included in the analysis. Following ablation of motor neurons, migration of neural crest cells were analyzed either by live imaging for 3–5 hours or by fixing embryo 3–5 hours post ablation. Fixed embryos were subsequently analyzed by in situ hybridization and immunohistochemistry as described above.

### Statistical Analysis

Chi Square and Fisher exact probably tests were performed (one tailed) for data presented in Figure 3D and Figure 4E.
